# Ultra-high-field magnetic resonance imaging reveals sex-specific recovery after ischemic stroke following treatment with three-dimensional human mesenchymal stem cell–derived extracellular vesicles

**DOI:** 10.7150/thno.129188

**Published:** 2026-06-17

**Authors:** Jamini Bhagu, Arshia Arbabian, Thurston Da Vitoria Lobo, Katherine Martinez, Dayna L. Richter, Hannah Bryant, Malathy Elumalai, Yan Li, Samuel C. Grant

**Affiliations:** 1 National High Magnetic Field Laboratory, Florida State University, Tallahassee, Florida, USA.; 2 Department of Chemical & Biomedical Engineering, FAMU-FSU College of Engineering, Florida State University, Tallahassee, Florida, USA.

**Keywords:** extracellular vesicles, mesenchymal stem cells, ischemic stroke, sodium MRI, diffusion MRI, sex differences, aging

## Abstract

Rationale: Ischemic stroke is sexually dimorphic. Biological sex can influence injury progression and response to treatment. Extracellular vesicles (EV) derived from three-dimensional (3D) human mesenchymal stem cell aggregates (3D-EV) are a promising candidate as a treatment, but their efficacy across these biological variables and *in vivo* behavior needs to be characterized. This study evaluated whether 3D-EV therapy enhances recovery following ischemic stroke in female and male models using ultra-high-field MRI and their influence on structural, ionic, and metabolic recovery.

Methods: A preclinical model of transient middle cerebral artery occlusion was used to longitudinally evaluate the efficacy of ultrasmall superparamagnetic iron oxide (USPIO)-labeled 3D-EV or saline at reperfusion through intra-arterial injection. MRI was performed at 21.1 T, which included T_2_-weighted, diffusion-weighted imaging, gradient-recalled echo imaging, and ²³Na chemical shift imaging. Proton magnetic resonance spectroscopy (¹H-MRS) was used to quantify changes in lactate, N-acetylaspartate, creatine, and choline within peri-infarct tissue. Imaging and behavioral outcomes were assessed over 21 days.

Results: USPIO-labeled 3D-EV resulted in localized hypointense contrast in the ischemic striatum, indicating delivery of treatment. T_2_-weighted MRI showed progressive lesion reduction, with a trend toward better recovery in females. ²³Na MRI revealed reduced sodium accumulation, with earlier ionic normalization in 3D-EV-treated animals. Diffusion recovery was observed with sex-dependent trajectories. ¹H-MRS showed lower lactate concentrations and preservation of other metabolites in EV-treated females. Behavioral differences were not significant.

Conclusions: 3D-EV therapy showed trends toward structural, ionic, and metabolic recovery following an ischemic insult. Ultra-high-field MRI and MRS can provide sensitive biomarkers to resolve these differences and support 3D-EV as a potential cell-free therapeutic candidate for ischemic stroke.

## Introduction

Stroke ranks as the third leading cause of death and long-term disability worldwide, with ischemic stroke accounting for nearly 70% of all cases. Its prevalence varies across regions and demographic groups (**Fig. S1A**) 1, with women experiencing higher stroke mortality than men in the United States (**Fig. S1B**). In recent years, stroke incidence among younger women (aged 18–35) has increased compared to age-matched men 2,3. These trends suggest that age and sex are biologically relevant factors of cerebrovascular vulnerability and recovery.

Currently, the only drug approved by food and drug administration (FDA) is thrombolytic, tissue plasminogen activator (TPA) that has limited treatment window of 4.5 h. Alternative interventions such as mechanical thrombectomy can extend this window up to 24 h. However, only small number of patients who meet strict imaging and clinical criteria are eligible 4,5. Of those receiving these treatments, only a small number of patients achieve full functional independence, emphasizing the need for adjunctive treatments that promote post-ischemic tissue repair. Women exhibit lower rates of functional independence even after adjusting for stroke severity 6-9. Some studies have reported that comorbidities, like diabetes, can have sex-specific outcomes 10. This suggests that biological differences contribute to recovery and underscore the need for including biological sex as a variable in preclinical research.

Hormonal exposure is a key biological factor influencing cerebrovascular vulnerability. Pregnancy, early postpartum period, and postmenopausal hormone therapy are associated with an increased risk of stroke 11-15. 17β-estradiol (E2), has been shown to play a neuroprotective role in various animal studies. These protective effects are attributed to several mechanisms, including the activation of neuroprotective signaling pathways and modulation of both local and systemic immune response following ischemic injury. However, clinical trials have not consistently demonstrated protective benefits. Together, these findings indicate that hormonal state modulates risk and recovery but doesn’t fully account for differences in outcomes across sex and age groups.

Ionic and metabolic disturbances evolve dynamically and may also vary across biological sex. ATP depletion, sodium–potassium ATPase failure, intracellular sodium accumulation, and lactate elevation during an ischemic attack leads to a disruption of cerebral metabolism 16. Advanced magnetic resonance techniques allow for *in vivo* quantification of these processes, providing measures of ionic imbalance, metabolic integrity, and structural injury that may clarify whether intrinsic differences in tissue recovery contribute to outcome disparities 17.

Mesenchymal stem cell-derived extracellular vesicles (MSC-EV) have recently emerged as potential candidate for post-ischemic tissue repair. Early MSC studies suggested therapeutic benefit, with a focus on paracrine mechanisms 18,19. Preclinical stroke models demonstrated that MSC-EV may reduce infarct volume, enhance functional recovery, and promote neurovascular remodeling across a range of experimental conditions. MSC-EV modulate multiple components of the post-ischemic microenvironment, including attenuating neuroinflammation, shifting microglial polarization toward reparative phenotypes, delivering neuroprotective microRNA cargo to support neuronal survival and synaptic plasticity 20-23, activating PI3K–AKT signaling pathways, suppressing NF-κB-mediated inflammation, enhancing angiogenesis through vascular endothelial growth factor (VEGF)-associated pathways, and preserving mitochondrial function under metabolic stress 24-26. These effects position MSC-EV as regulators of inflammatory, metabolic, and structural repair following stroke.

*In vitro* analysis of the EV under 3D conditions demonstrated a significant increase in expression of neuroprotective and anti-apoptotic miRNA highlighting their potential for neurological therapies 27. The hypoxic microenvironment that develops within the core of 3D spheroids likely contributes to this enhanced therapeutic potency. This is consistent with evidence that hypoxic preconditioning and matrix-bound nanovesicle approaches improve angiogenic and metabolic signaling in MSC-EV 28,29. Collectively, these findings suggest that 3D aggregate cultures can enhance the biogenesis of therapeutic MSC-EV supporting both translational research and clinical biomanufacturing.

The present study primarily evaluates 3D-EV in young female and age-matched male rodents using multimodal ultra-high-field MRI to characterize longitudinal changes in lesion volume, tissue diffusivity, ionic homeostasis, and metabolic integrity following stroke. Our hypothesis is that EV derived from 3D human MSC aggregates (3D-EV) therapy provides improved tissue recovery and energy homeostasis with intrinsic differences across biological sex.

## Materials and Methods

### Dynamic Aggregation of MSC

Human bone marrow MSC were obtained from RoosterBio Inc. (Frederick, MD) and expanded in complete culture medium (CCM) consisting of minimum essential medium-alpha (α-MEM) supplemented with 10% fetal bovine serum (FBS, Atlanta Biologicals, Lawrenceville, GA) and 1% penicillin–streptomycin (Life Technologies, Carlsbad, CA) at 37 °C in 5% CO₂. Cells were passaged at 70–80% confluence using 0.25% trypsin–EDTA and reseeded at 2,000 cells/cm², with experiments conducted at passages 5–6. To generate 3D aggregates, 200,000 cells were seeded into ultra-low-attachment six-well plates (Corning, NY) and incubated under dynamic conditions using a WAVE Bioreactor (8°, 20 cycles/min) for 12 h, facilitating spontaneous aggregation into compact spheroids of 100–300 µm in diameter 30. Unless otherwise noted, all reagents were obtained from Sigma-Aldrich (St. Louis, MO).

### EV Isolation and Labeling with Ultrasmall Superparamagnetic Iron Oxides (USPIO)

Culture medium was replaced with EV-depleted CCM and conditioned media were collected after three days. 3D-EV were isolated using a polyethylene glycol-based enrichment method (ExtraPEG) followed by differential ultracentrifugation 31. Briefly, media were centrifuged sequentially at 500 g (5 min), 2,000 g (10 min), and 10,000 g (30 min) to remove cells, apoptotic bodies, and large vesicles, respectively. The clarified supernatant was mixed 1:1 with 16% (w/v) PEG-6000 in 1 M NaCl, incubated overnight at 4°C, and centrifuged at 3,200 g for 1 h to pellet crude EVs. The pellet was resuspended in PBS and further purified by ultracentrifugation at 100,000 g for 70 min at 4 °C, then stored at -80 °C until use.

For labeling, USPIO nanoparticles (Fe₃O₄; average diameter ≈ 5 nm; 5mg/mL in water; catalog #725331-5ML, Sigma-Aldrich, St. Louis, MO, USA) were diluted with purified 3D-EV to a final concentration of 0.1–0.5 mg/mL in a total volume of 500 µL. USPIO were incorporated using probe sonication (Fisher Scientific, Hampton, NH, USA) in 3 cycles of 2-second bursts for 10 s on ice. The EV–USPIO mixture was then subjected to an additional round of ExtraPEG purification to remove unbound nanoparticles and resuspended in 50 µL saline.

### Nanoparticle Tracking Analysis

Nanoparticle tracking analysis (NTA) was used to assess the size distribution and particle concentration of isolated 3D-EV. Samples were analyzed on the NanoSight LM10 system (Malvern Panalytical, Salisbury, UK) in triplicates using 60-second video recordings. Merged datasets were used to calculate mean, mode, and percentile diameters (D10, D50, D90) and total particle concentration.

### Transmission Electron Microscopy (TEM)

USPIO-labeled EV were resuspended in 50–100 μL of sterile-filtered PBS to maintain particle stability. 5 μL of EV were carefully pipetted onto Parafilm to prevent excess sample loss. A carbon-coated 400 Hex Mesh Copper grid (Electron Microscopy Sciences, EMS) was then placed coating side down onto each EV droplet using fine-tip forceps and incubated for one hour at room temperature to allow absorption. Following incubation, grids were washed three times with sterile-filtered PBS and samples were fixed in 2% paraformaldehyde (PFA, EM Grade) for 10 min. After fixation, grids were transferred onto a 20-μL drop of 2.5% glutaraldehyde (EM Grade) and incubated for an additional 10 min to enhance structural stability. The samples were negatively stained using 2% uranyl acetate (EMS grade) for 10 min. Grids were incubated in 0.13% methyl cellulose combined with 0.4% uranyl acetate for 10 min to further stabilize and embed the EV. The coated side of the grids was left to air dry before imaging using the Hitachi HT7800 electron microscope housed at Florida State University.

### Inductively Coupled Plasma Mass Spectrometry

To quantify USPIO incorporation efficiency, iron content of labeled and unlabeled 3D-EV preparations was measured by high-resolution inductively coupled plasma mass spectrometry (ICP-MS) (Finnigan Mat ELEMENT1, Waltham, MA). 3D-EV samples were dissolved in 1 mL of 7 N nitric acid and dried at 140°C, then redissolved in 1 mL of 2% nitric acid with 15 µL indium as an internal standard. Solutions were introduced via a 100 µL nebulizer and PFAS Teflon spray chamber at a plasma power of 1200 W. Iron (⁵⁶Fe) was measured at intermediate resolution (2500 amu) at the low-mass side of the peak to avoid overlap with the ⁴⁰Ar¹⁶O interference. Indium signal was used to correct plasma drift. Calibration was performed using 0.5, 1, and 10 ppb iron standards in 2% nitric acid (r² = 0.99). Iron concentration was normalized to particle count determined by NTA to yield iron content per EV.

### Animal Model

All animal experiments were conducted in accordance with the guidelines of the Animal Care and Use Committee (ACUC) at Florida State University and complied with the United States Public Health Service Policy on Humane Care and Use of Laboratory Animals and the NIH Guide for the Care and Use of Laboratory Animals (NIH Publications, No. 8023, 1978 revision). Transient focal cerebral ischemia was induced using the middle cerebral artery occlusion (MCAO) method described by Longa et al. Briefly, a silicone-coated monofilament (Doccol Corp., Sharon, MA, USA) was advanced through the external carotid artery into the internal carotid artery to occlude the origin of the middle cerebral artery 32. After one hour occlusion, the filament was withdrawn to allow reperfusion. Animals were randomized to treatment groups immediately following reperfusion and prior to imaging confirmation of stroke.

A total of 33 adult Sprague–Dawley rats (23 females, 10 males, 200–250 g; Envigo, Indianapolis, IN, USA) were enrolled in the study. Treatments were administered as a single intra-arterial injection to the internal carotid artery immediately following reperfusion. Five females and three males received a dose of approximately 10 billion USPIO-labeled 3D-EV in 50 µL, while five females and four males received 50 µL of saline as vehicle control.

Two exclusion criteria were applied uniformly across all animals regardless of treatment group assignment. First, animals were excluded if they failed to present with a striatal infarct on Day 1 post-surgery MRI, confirming unsuccessful stroke induction. Secondly, as a pre-specified humane endpoint, animals were removed from the study and humanely euthanized if body weight declined more than 20% from baseline at any point during the post-operative period. All surviving animals that met both inclusion criteria were retained through the study endpoint. The number of animals excluded or lost to each criterion is reported in the Results section.

Anesthesia was induced with 4–5% isoflurane in oxygen and maintained at 1–3% for the duration of the procedure. All animals received preoperative bupivacaine and buprenorphine for analgesia and sterile saline for hydration. Animals recovered in a temperature-controlled incubator before being returned to their home cages. Additional postoperative buprenorphine was administered as needed.

### *In Vivo* Magnetic Resonance Imaging and Spectroscopy

All *in vivo* imaging was performed on a 21.1 T (900 MHz) vertical-bore MRI scanner (National High Magnetic Field Laboratory, Tallahassee, FL, USA) equipped with a Bruker Neo console and ParaVision 360.3.2-5 software. Animals were positioned supine in a custom-built (33 mm inner diameter), double-tuned ^23^Na/^1^H birdcage radiofrequency (RF) coil tuned to 238 MHz and 900 MHz, respectively. Isoflurane anesthesia was induced at 4–5% and maintained at 2–3% in 100% O₂ throughout scanning. Core temperature was stabilized using the gradient chiller airflow system, and respiration was continuously monitored (Small Animal Instruments, Inc., Stony Brook, NY, USA) and used for acquisition triggering.

Anatomical localization images in the axial, coronal, and sagittal planes were obtained using ^1^H rapid acquisition with relaxation enhancement (RARE) sequence 33. T₂-weighted (T_2_W) images were acquired with a ^1^H 2D RARE fast spin-echo sequence (TE/TR = 25/6000 ms) employing fat suppression and respiratory gating, with 23 slices (0.5 mm thickness) and 100 × 100 µm in-plane resolution.

The diffusion weighted 2D echo-planar images DWI-EPI) were acquired at (TE/TR = 20/5000 ms) 200 × 200 µm in-plane resolution and 1 mm slice thickness. The diffusion gradient duration (δ) and separation (Δ) were 3 ms and 11 ms, respectively. Five b-values of 0, 400, 800, 1200, and 1900 s/mm² were used to generate apparent diffusion coefficient (ADC) maps, which quantify the magnitude of water diffusion within tissue.

^1^H MRS was acquired using a single-shot, relaxation-enhanced, semi-adiabatic localization by adiabatic refocusing (sLASER) from a 3 mm³ voxel 34,35. Anatomical MRI was used as an anatomical reference to ensure reproducible positioning. A 4 kHz bandwidth excitation pulse was applied to improve metabolite detection while minimizing water signal. Metabolites of interest include lactate, creatine, choline, and N-acetylaspartate. Water suppression was performed using variable power radiofrequency pulses with optimized relaxation delays (VAPOR).

An external metabolite reference phantom based on the Braino 2.0 formulation was used for calibration, prepared as previously described 36. The phantom contained 6.5 mM N-acetyl-L-aspartic acid, 0.5 mM choline chloride, 8 mM creatine monohydrate, 2 mM myo-inositol, 4.0 mM L-glutamic acid, 3 mM sodium lactate, 2.5 mM L-phenylalanine, 1 mM L-alanine, 2 mM γ-aminobutyric acid, 3.5 mM taurine, 1 mM L-aspartic acid, 0.5 mM sodium succinate, 1 mM trimethylsilylpropanoic acid (TSP, 1.0 mM) as a chemical shift reference, 50 mM potassium phosphate monobasic as buffer, 0.5 mM gadodiamide to adjust relaxation properties, and 1 mM sodium azide as a preservative. The phantom composition and preparation follow the Braino 2.0 protocol described previously 36.

To assess the* in vivo* biodistribution of USPIO-labeled 3D-EV, a ^1^H gradient-recalled echo (GRE) sequence was used (TE/TR = 4/1000 ms; 50 × 50 µm in-plane resolution; 0.5 mm slice thickness). Signal intensity was quantified using manually defined regions of interest (ROIs) placed over the ischemic striatum and the anatomically matched contralateral region.

3D chemical shift imaging is a multi-voxel spectroscopic imaging technique that uses non-selective RF-pulses with sampling of a free induction decay (FID) after each phase encoding step. Sodium imaging was conducted using a 3D ^23^Na chemical shift imaging (CSI) sequence (TE/TR = min/60ms; 1 mm isotropic resolution) with non-selective RF pulses and FID sampling after each phase-encoding step 37.

### Image and Spectral Processing

Segmentation and quantitative analyses for ^23^Na CSI, T₂-weighted, DWI-EPI, and GRE datasets were conducted using AMIRA 3D visualization software (Thermo Fisher Scientific, Waltham, Massachusetts). ^23^Na CSI data were reconstructed in MATLAB (MathWorks, Natick, MA, USA) and zero-filled to 0.5 mm isotropic resolution. The ischemic lesion was defined using a threshold, which is set by using the mean and standard deviation of the signal in the contralateral hemisphere (mean ± 2 SD). Day 1 datasets served as reference volumes for longitudinal registration across days 0, 3, 7, and 21. ADC maps were generated from diffusion data using the same thresholding approach. Quantitative changes in ADC within the lesion were tracked across the 21-day recovery period. All ^1^H-MRS spectra were processed in TOPSPIN 4.1.4 (Bruker). Metabolite peaks corresponding to lactate (1.3 ppm), N-acetylaspartate (2.0 ppm), creatine (3.0 ppm), and choline (3.2 ppm) were normalized to an external phantom.

### Neuromotor Analysis

Behavioral performance was evaluated at baseline (day 0, prior to MCAO) and on postoperative days 3, 7, 14, and 21, with all assessments conducted before MRI scanning to minimize animal use. All tests were performed under double-blinded conditions, and the order of testing was randomized. A rest interval of approximately 10 min was provided between individual tests to reduce fatigue and stress.

Forelimb use asymmetry was assessed using the cylinder rearing test as previously described 38. Each rat was placed in a transparent Plexiglas cylinder and allowed to freely explore for up to 10 min. During vertical exploration, spontaneous wall contacts made independently with the right, left, or both forelimbs were recorded. A total of 20 wall placements were analyzed per animal, and the percentage of right, left, and bilateral contacts was calculated. Animals exhibiting unilateral ischemic injury demonstrated a preference for one forelimb, reflected in an asymmetry index that served as a measure of motor deficit and recovery.

Anxiety-like behavior was evaluated using the elevated plus maze 39 consisting of two open and two enclosed arms elevated 52 cm above the floor. Each animal was placed in the central zone facing an open arm and allowed to explore for 5 min while being recorded with a video camera. The number of entries and total time spent in the open versus closed arms were quantified as indicators of anxiety and post-stroke behavioral recovery.

Locomotor activity and exploratory behavior were examined using the open field test 40. Rats were placed in the center of an open square arena (92 × 92 × 56 cm) divided into 36 equal grid squares and recorded for 10 min. Total distance traveled and the duration spent in the central region versus the periphery were analyzed using MATLAB (MathWorks, Natick, MA, USA). These measures were used to evaluate both spontaneous locomotion and anxiety-like behavior.

### Statistical Analysis

All statistical measures were analyzed using a generalized linear mixed-effects model (GLMM) with a Gaussian distribution in JMP Pro 19 (SAS Institute Inc., Cary, NC). The fixed effects were Day, Treatment, and their interaction (Day × Treatment). Post hoc pairwise comparisons for the Treatment × Day interaction were conducted using Student's t-test applied to least squares means (LS Means) derived directly from the GLMM.

Normality of residuals was confirmed for each response variable using the Shapiro–Wilk test (all p > 0.05), and homogeneity of variance was verified using Levene's test (all p > 0.05). Each variable was analyzed independently. No correction for multiple comparisons was applied across outcome variables. Pairwise LS Means comparisons were performed without adjustment. This approach was chosen to leverage the sensitivity to potential treatment-related signals, given the modest sample size, while acknowledging the increased risk of Type I error inherent to unadjusted multiple comparisons.

## Results

### Successful Delivery of USPIO-Labeled 3D-EV to the Ischemic Striatum

3D MSC aggregates were formed in suspension compared to conventional 2D cultures for EV isolation (**Fig. S2**). NTA demonstrated that the EV size was centered around ~120 nm, consistent with small extracellular vesicles known as exosomes (30-200 nm) (**Fig. S3**). Transmission electron microscopy (TEM) confirmed the typical exosomal morphology of USPIO-labeled EV (**Fig. S4**). ICP-MS confirmed successful incorporation of USPIO into the 3D-EV. The unlabeled 3D-EV contained a mean iron content of 3.01×10⁻⁸ pg Fe/EV, while USPIO-labeled 3D-EV had a mean iron content of 9.80×10⁻⁷ pg Fe/EV, an approximately 32-fold increase (**Table 1**). These results confirm that USPIO labeling resulted in meaningful nanoparticle incorporation into the EV rather than reflecting endogenous iron alone.

Together, the NTA, TEM, and ICP-MS data presented here confirm that the material used in the present study is structurally and compositionally consistent with prior preparations from the same 3D aggregate culture system and isolation workflow 27,41,42. Canonical EV protein marker profiling was performed on EVs derived from the same production system in our prior publications 27; while this was not repeated on the present preparation, the convergent morphological and physicochemical characterization supports attribution of the observed *in vivo* effects to the EV fraction.

A total of 33 adult Sprague–Dawley rats (23 females, 10 males) were enrolled across the treatment groups. Of these, three animals died during surgical recovery (one female and two males). Three were euthanized due to post-operative body weight loss exceeding 20%, and ten (nine females and one male) were excluded because of the absence of a confirmed striatal infarct on Day 1 MRI. This resulted in a final female cohort of ten animals (five 3D-EV treated, five saline control) and a final male cohort of seven animals (three 3D-EV treated, four saline control). The higher rate of stroke induction failure in females (39%) compared to males (10%) likely reflects the neuroprotective influence of circulating estrogen and progesterone, which are well documented to reduce infarct susceptibility in female rodents 43-45.

**Figure 1:**
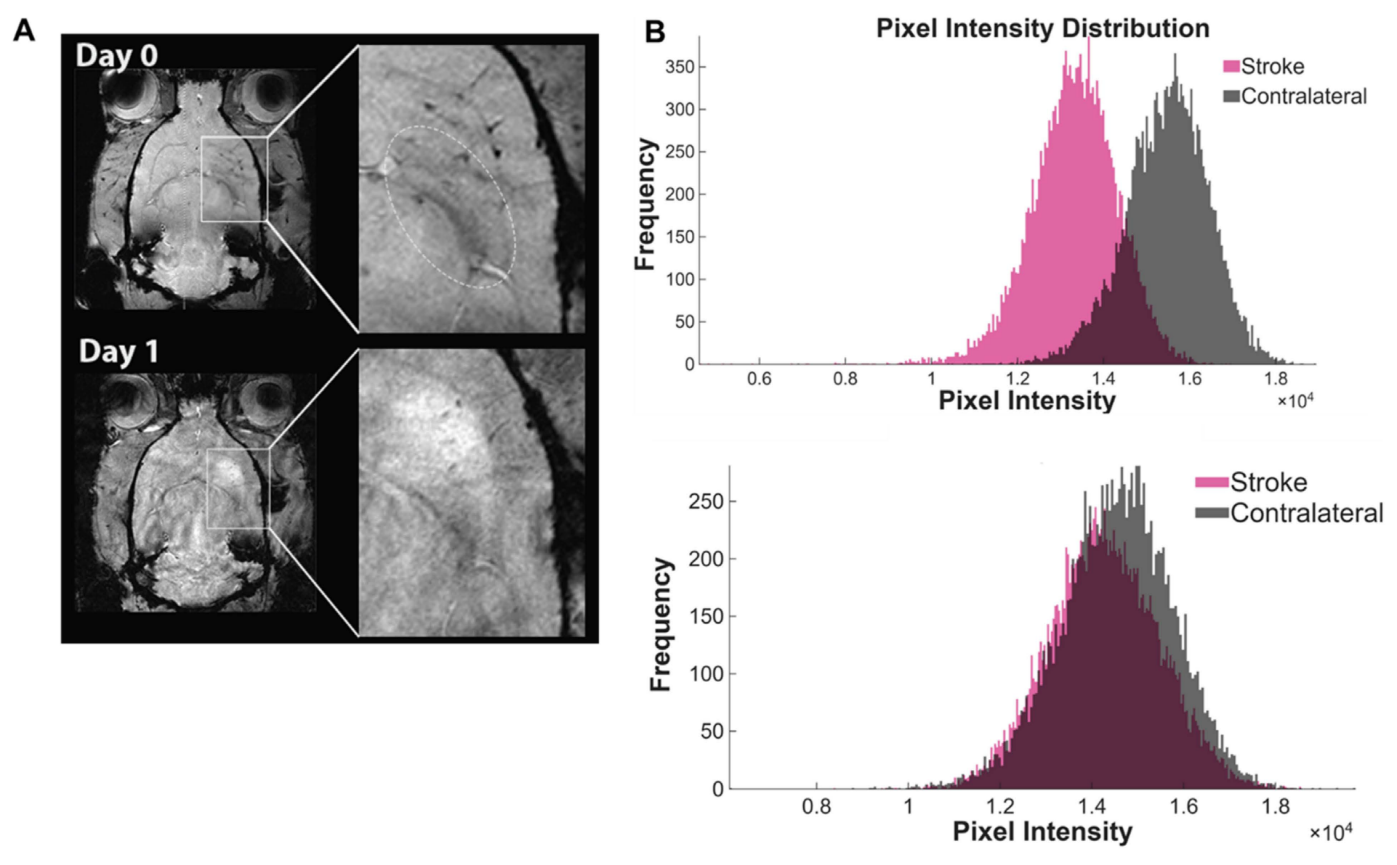
** Biodistribution of USPIO –Labeled EV Following Intra-arterial Administration.** (A) GRE images of a representative animal administered USPIO only on days 0 (top) and 1 (bottom). Boxes indicate magnified striatal region in the ischemic hemisphere. (B) Pixel intensity distributions of the stroke (pink) and contralateral (gray) hemispheres at Day 0 (top) and Day 1 (bottom).

Following transient ischemic occlusion, *in vivo* MRI was performed approximately 3 h after reperfusion to verify delivery of USPIO-labeled 3D-EV 17,29,41. Animals that received the 3D-EV treatment showed localized hypointense contrast within the left striatum, indicating EV accumulation in the ischemic territory (**Fig. 1A**). On day 0, the mean signal intensity in the ipsilateral hemisphere was lower than in the contralateral side, reflecting the presence of USPIO. By day 1, the ipsilateral hemisphere became hyperintense relative to the contralateral side, indicating both the expected evolution of ischemic signal and clearance of USPIO contrast. The voxel-intensity distribution broadened from day 0 to day 1, consistent with increasing heterogeneity of the evolving lesion (**Fig. 1B**).

### Combined ²³Na CSI and ¹H MRI Reveal Sex-Dependent Ionic and Structural Recovery Following 3D-EV Therapy in Ischemic Stroke

**Figure 2:**
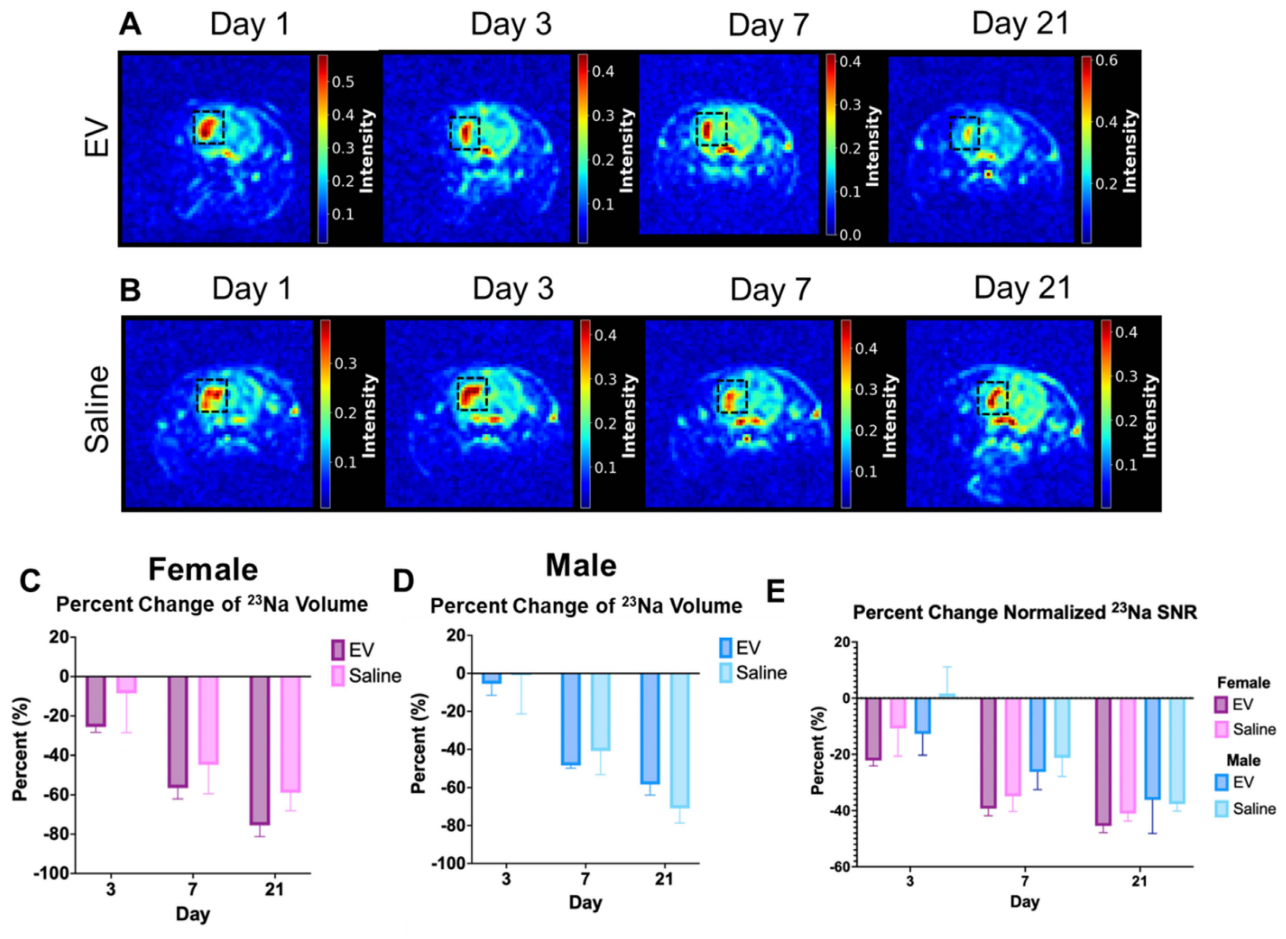
**Longitudinal Changes in ²³Na MRI Signal Following EV or Saline Treatment.** (A,B) Reference female EV and control animals on days 1, 3, 7, and 21 post-MCAO. The black square delineates the ischemic lesion region within the stroke hemisphere. (C,D) Percent change in sodium lesion volume in females and males. (E) Percent changes of males and females combined. Statistical significance was assessed using a GLMM with Student's t-test for post hoc pairwise comparisons. No correction for multiple comparisons across outcome measures was applied. *p < 0.05.

Sodium MRI is a sensitive metric for assessing tissue recovery following ischemic stroke. The temporal progression of ischemic injury in female and male animals treated with 3D-EV or saline control was evaluated over a 21-day recovery period (**Fig. 2**). Representative ²³Na maps from 3D-EV-treated and saline-treated female animals across Days 1, 3, 7, and 21 demonstrated progressive reduction in lesion-associated sodium signal in both groups over the observation period (**Fig. 2A, 2B**). Representative ²³Na maps from male 3D-EV-treated and saline-treated animals are shown in **Fig. S5**. By day 3, 3D-EV-treated animals of both sexes exhibited smaller sodium-defined lesion volumes compared with their corresponding saline controls. In both females and males, lesion volume continued to decline significantly through day 21 (**Fig. 2C, 2D**). No statistically significant difference in lesion volume reduction was observed between 3D-EV-treated females and males. However, the females showed a greater reduction at several timepoints (**Fig. 2D**). Saline-treated females and males displayed similar overall reductions, with greater variability observed at later timepoints.

**Figure 3:**
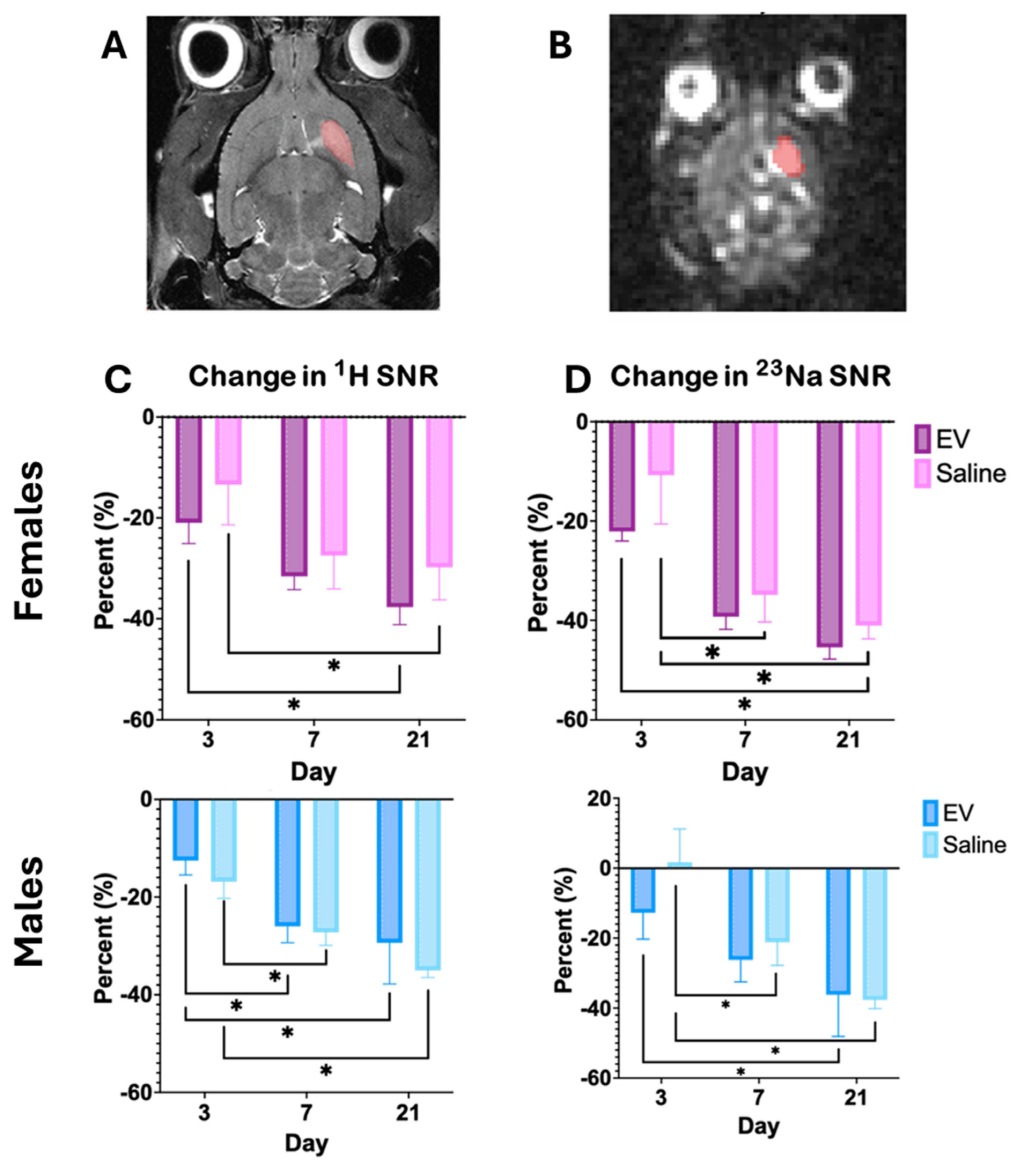
**Longitudinal Changes in SNR in T_2_W and Sodium MRI.** (A) T_2_W and (B) sodium image of a representative female animal on Day 1 used as the reference image for registration. Highlighted volume was used as reference volume to measure the changes in SNR. (C) & (D) demonstrates the percent change in SNR within the same volume as day 1. Statistical significance was assessed using a GLMM with Student's t-test for post hoc pairwise comparisons. No correction for multiple comparisons across outcome measures was applied. *p < 0.05.

To evaluate signal intensity changes within a fixed reference volume, percent change in SNR was tracked longitudinally within the Day 1 lesion volume for both T_2_-weighted and ²³Na imaging (**Fig. 3A, 3B**). In female animals, neither T_2_W nor ²³Na SNR showed significant differences between 3D-EV and saline treatment groups across Days 3, 7, and 21 (**Fig. 3C**). Similarly, no significant between-group differences were detected in male animals across the same timepoints (**Fig. 3D**). These findings suggest that while overall lesion volumes changed over time, SNR changes within the fixed reference volume did not differ significantly as a function of treatment in either sex.

**Figure 4:**
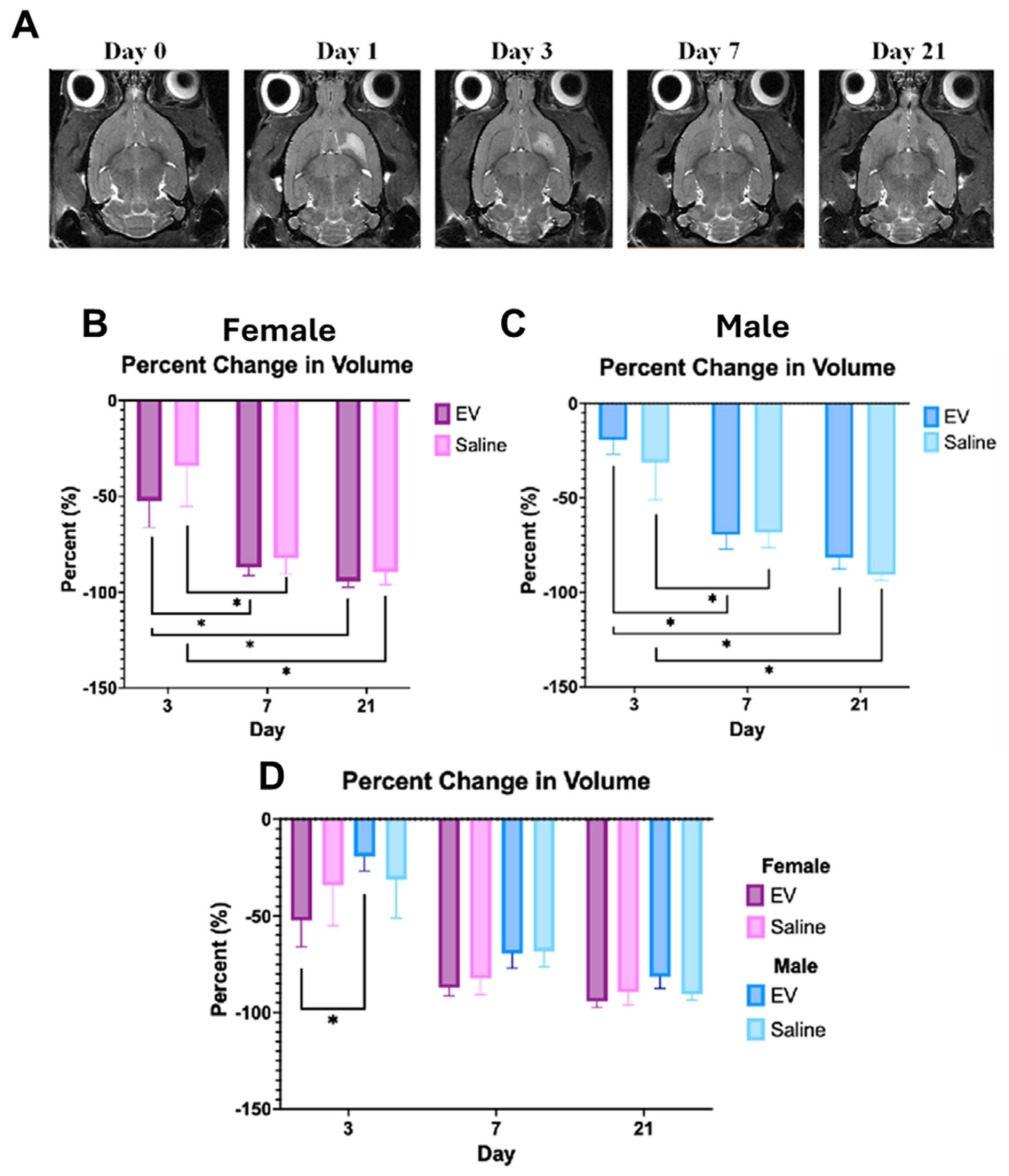
** Longitudinal Changes in Ischemic Lesion Volume Following EV or Saline Treatment.** (A) Representative T_2_-weighted MRI images from a female animal at Days 0, 1, 3, 7, and 21 post-stroke. The hyperintense signal in the striatal region delineates the ischemic lesion; progressive reduction in signal intensity reflects lesion volume contraction over time. Percent change in lesion volume relative to Day 1 in (B) females and (C) males treated with EV or saline, showing a similar pattern of EV-mediated lesion reduction. (D) Sex-stratified comparison of lesion volume changes across treatment groups at Days 3, 7, and 21. Statistical significance was assessed using a GLMM with Student's t-test for post hoc pairwise comparisons. No correction for multiple comparisons across outcome measures was applied. *p < 0.05.

The initial ischemic injury was confirmed 24 h post-reperfusion using ^1^H T_2_W imaging in all animals (**Fig. 4A**). Although between-group differences did not reach statistical significance, 3D-EV-treated females demonstrated a consistent trend toward smaller lesion volumes compared to saline controls across the 21 day period, a pattern less evident in male animals (**Fig. 4B, 4C, 4D**). Across all groups, T_2_-defined lesion volume declined over the early recovery period. Notably, at Day 3, 3D-EV-treated females exhibited significantly smaller lesion volumes than 3D-EV-treated males, suggesting a possible sex-dependent difference in the early trajectory of EV-mediated lesion resolution (**Fig. 4B, 4C**).

### Diffusion MRI Reveals Heterogeneous Diffusion Recovery Following 3D-EV Treatment After Ischemic Stroke

**Figure 5:**
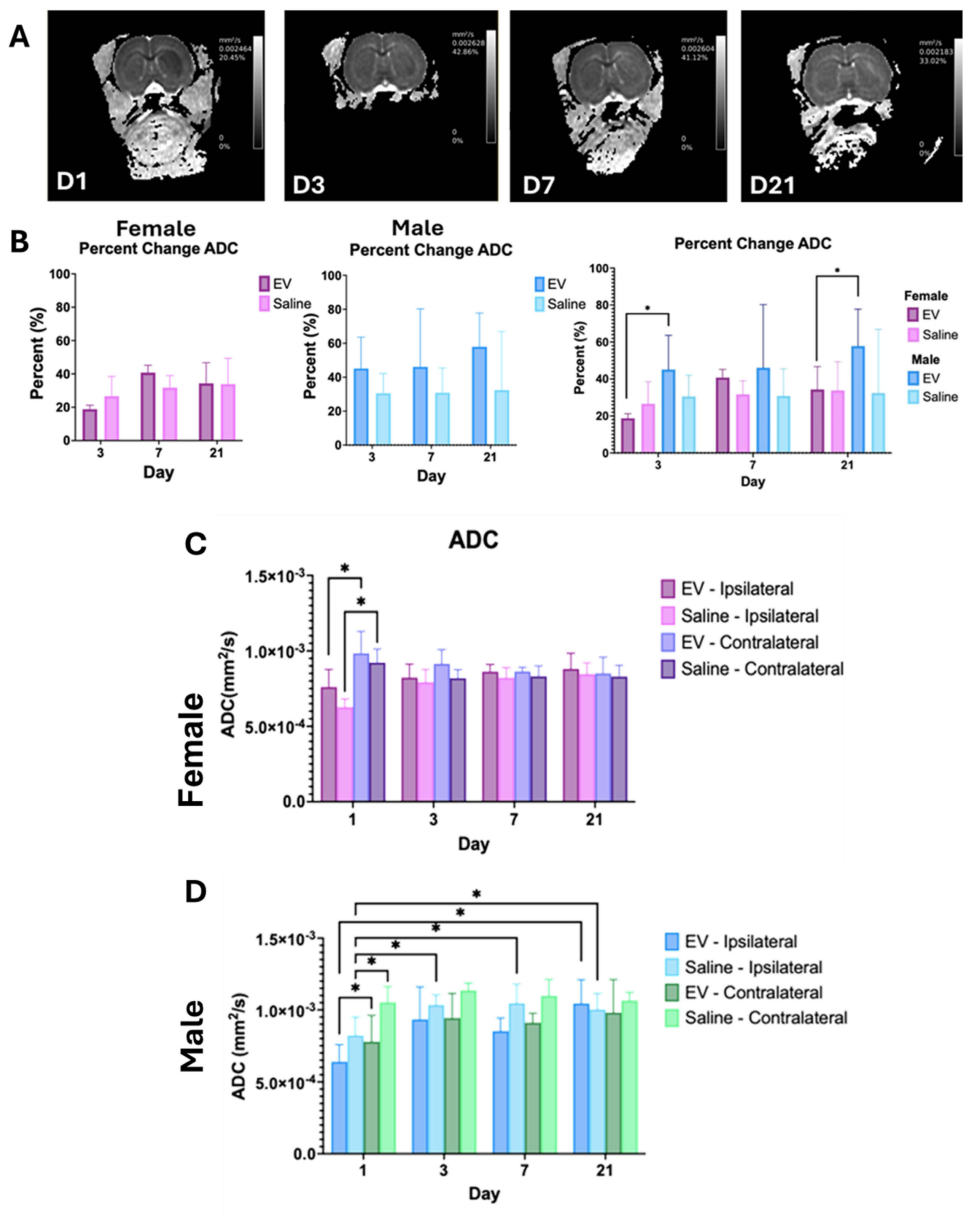
** Longitudinal Changes in Apparent Diffusion Coefficient Following EV or Saline Treatment.** (A) Representative ADC maps from a single animal at Days 1, 3, 7, and 21 post-MCAO. ADC values (mm²/s) and the percentage of pixels within the displayed range are indicated in the upper right corner of each image. (B) Percent change in ADC relative to Day 1 in female (left) and male (middle) animals treated with EV or saline, and in a combined sex comparison (right). (C) Absolute ADC values (mm²/s) in the ipsilateral (ischemic) and contralateral hemispheres of female and (D) male animals treated with EV or saline across Days 1, 3, 7, and 21. Statistical significance was assessed using a GLMM with Student's t-test for post hoc pairwise comparisons. No correction for multiple comparisons across outcome measures was applied. *p < 0.05.

DWI and ADC mapping were performed to characterize changes in tissue water diffusion across the 21-day recovery period. ADC maps demonstrated progressive changes in diffusion from Day 1 through Day 21, with ADC values increasing over time (**Fig. 5A**). Percent change in ADC relative to Day 1 did not vary significantly between EV and saline groups in females or males individually (**Fig. 5B**). However, significant differences between males and females were observed at Days 3 and 21, with male animals demonstrating a greater recovery of ADC (**Fig. 5B**).

Further analysis of ADC maps revealed differences in diffusivity between hemispheres (**Fig. 5C, 5D**). A significant decrease in ADC was observed in the ipsilateral hemisphere on day 1. This is consistent with acute cytotoxic edema commonly associated with ischemia. The ADC values increased in both the control and treated female groups after day 1. Although not statistically significant, the ADC was trending toward levels comparable to those on the contralateral side (**Table 2**). In males, the 3D-EV-treated group exhibited a significant elevation in ADC by day 21. By this time point, diffusivity differences between hemispheres were minimal.

### ^1^H-MRS Uncovers Preserved Neuronal Metabolism and Energetic Recovery After 3D-EV Treatment

Proton MRS was performed to assess longitudinal changes in metabolite concentrations within the ischemic hemisphere. Representative spectra were acquired from (3 mm)³ voxel placed in the ipsilateral and contralateral hemispheres to quantify key metabolites, including lactate (Lac, 1.3 ppm), N-acetylaspartate (NAA, 2.0 ppm), creatine (Cre, 3.0 ppm), and choline (Cho, 3.2 ppm).

**Figure 6:**
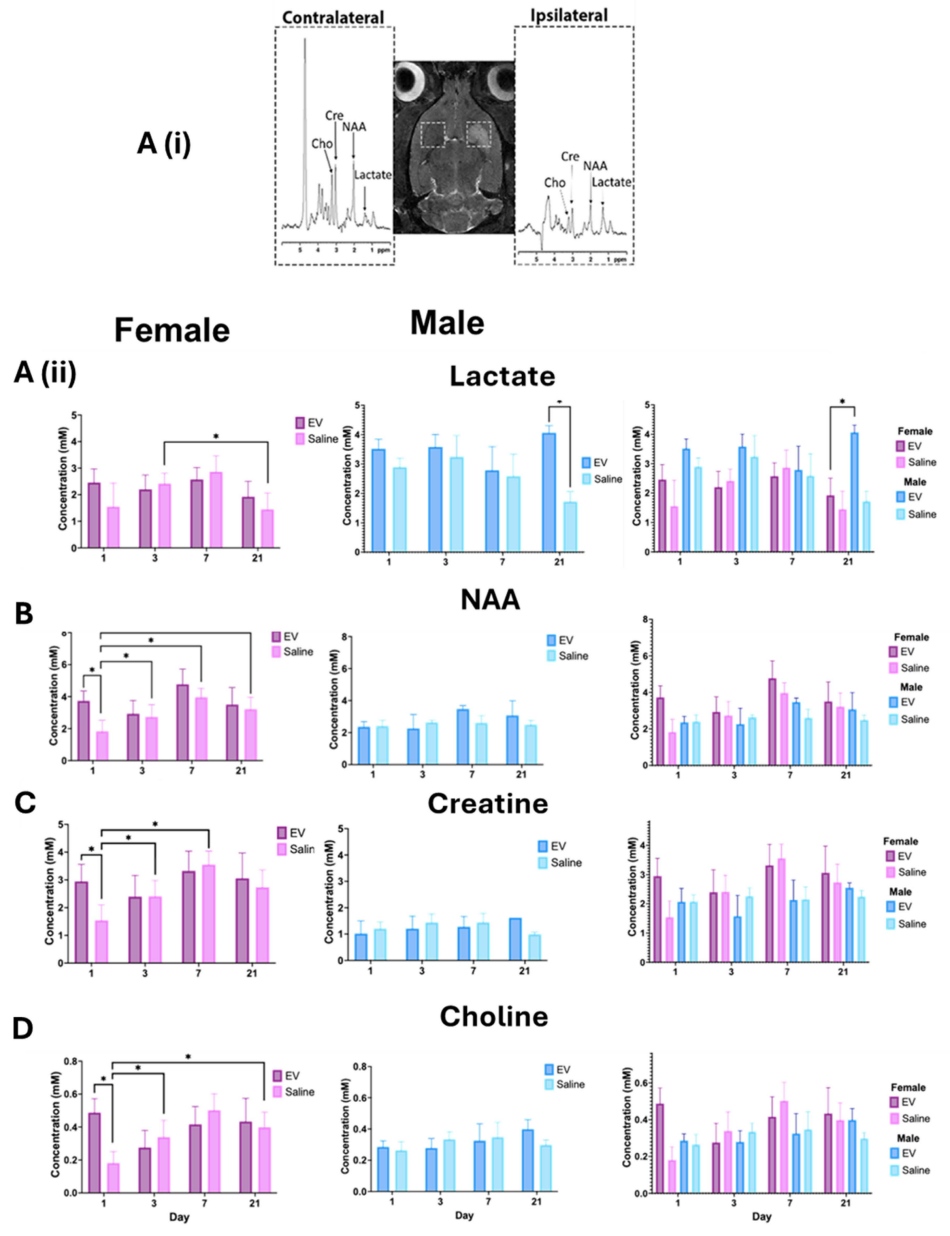
** Longitudinal ¹H MRS Metabolite Concentrations in the Ischemic and Contralateral Hemispheres Following EV or Saline Treatment. (A, top)** Representative T_2_-weighted MRI from a female saline-treated animal on Day 1, illustrating bilateral voxel placement (dashed boxes) in the ipsilateral (ischemic) and contralateral striatum used for spectroscopic acquisition. Flanking spectra show representative ¹H MRS signals from the contralateral (left) and ipsilateral (right) hemispheres, with labeled peaks for choline (Cho), creatine (Cre), N-acetylaspartate (NAA), and lactate. Line broadening of 10 Hz was applied for display. Longitudinal concentrations of **(A)** lactate, **(B)** NAA, **(C)** total creatine, and **(D)** choline are shown for female (left, purple/pink), male (middle, blue), and combined sex (right) treatment groups across Days 1, 3, 7, and 21 post-MCAO. Data are presented as mean ± SEM. Statistical significance was assessed using a GLMM with Student's t-test for post hoc pairwise comparisons. No correction for multiple comparisons across outcome measures was applied. *p < 0.05.

Lactate concentration increased in all animals following ischemia, suggesting a shift toward anaerobic metabolism (**Fig. 6A**). Interestingly, both the control and 3D-EV treated female groups exhibited lower lactate levels than males. However, the sex comparisons were not statistically significant. The 3D-EV-treated female group showed higher concentrations of NAA, total creatine, and choline 24 h post-reperfusion. These metabolite levels remained relatively stable through day 21, though between-group differences at this timepoint did not reach statistical significance. In contrast, the female saline group showed significantly lower metabolite concentrations 24 h post-reperfusion but exhibited partial recovery by day 21 (**Fig. 6B, 6C, 6D**). Male cohorts showed lower NAA, creatine, and choline concentrations relative to females, though no statistically significant longitudinal changes were observed over the 21-day period.

### 3D-EV Treatment Dynamically Alters Exploratory Behavior and Motor Coordination During Post-Stroke Recovery

To evaluate behavioral recovery and anxiety-related performance following ischemic injury, rats were reassessed using three standardized behavioral paradigms: the open field test, the elevated plus maze test, and the cylinder rearing test.

**Figure 7:**
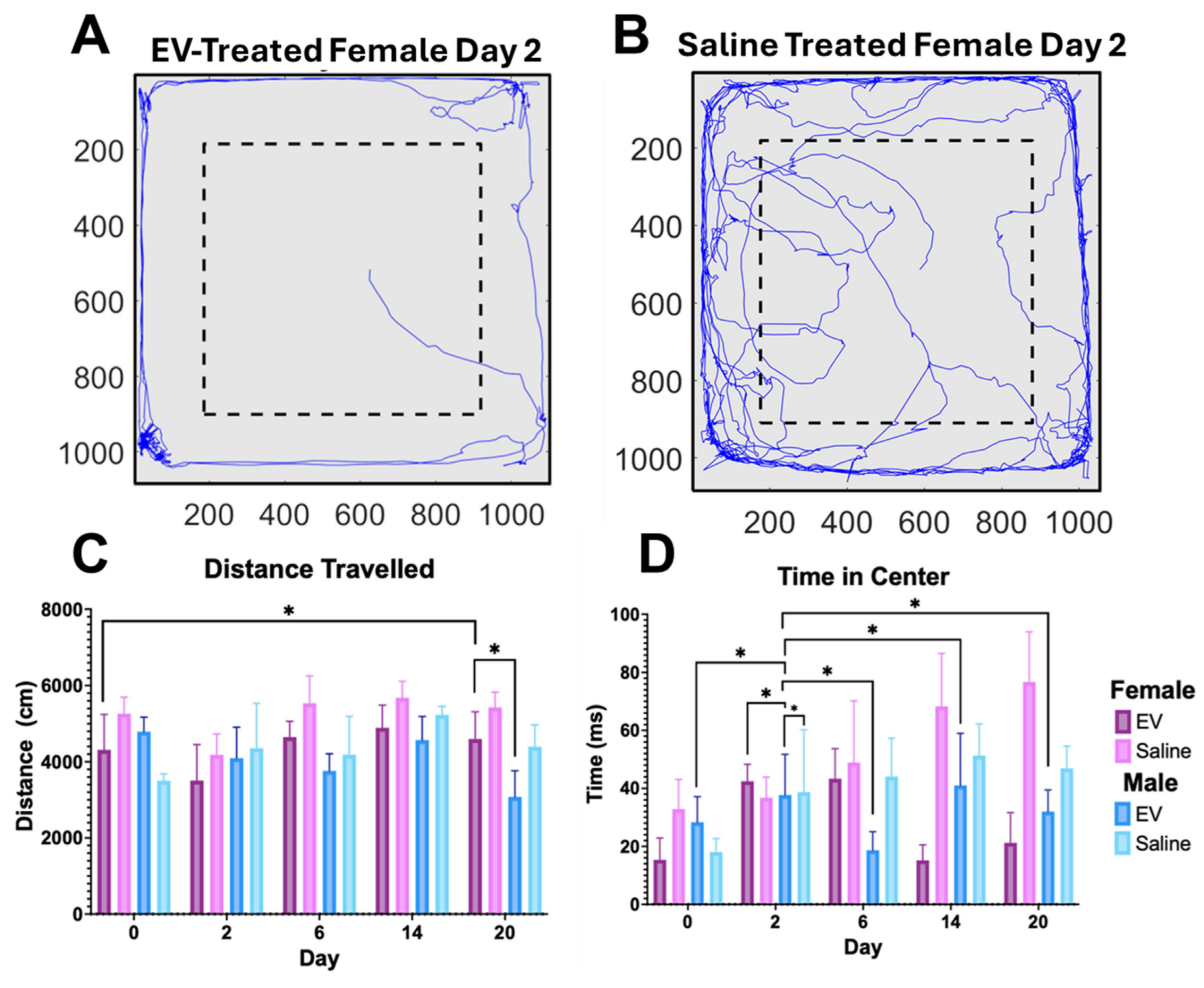
** Open Field Test Assessment of Locomotor Activity and Anxiety-Like Behavior Following EV or Saline Treatment.** (A) Representative open field movement trajectories from an 3D-EV-treated (left) and saline-treated control (right) animal. The blue line traces the animal's movement path over the duration of the test session. The dashed box delineates the center zone. (B) Total distance travelled (left) and time spent in the center zone (right) across Days 0, 2, 6, 14, and 20 post-MCAO, stratified by sex and treatment group. Statistical significance was assessed using a GLMM with Student's t-test for post hoc pairwise comparisons. No correction for multiple comparisons across outcome measures was applied. *p < 0.05.

The open field test was used to assess locomotor activity and exploratory behavior in animals. Movement trajectories illustrated differences in exploratory patterns between treatment groups (**Fig. 7A, 7B**). The 3D-EV treated animals spent more time in the peripheral zone, consistent with thigmotaxis. At baseline, EV-treated animals demonstrated similar exploratory behavior, with reduced time in the center. In contrast, the saline-treated animals exhibited greater exploration of the field, including the center zone. It is worth noting that animals across all groups had traveled comparable total distances (**Fig. 7C, 7D**), suggesting that locomotor ability was preserved. By day 6, 3D-EV-treated females spent more time in the center, which may indicate reduced anxiety (**Fig. 7D**). Both male treatment groups showed increased time in the center on day 2. Locomotor activity remained stable across sex and treatment groups, confirming that EV administration did not impair mobility.

**Figure 8:**
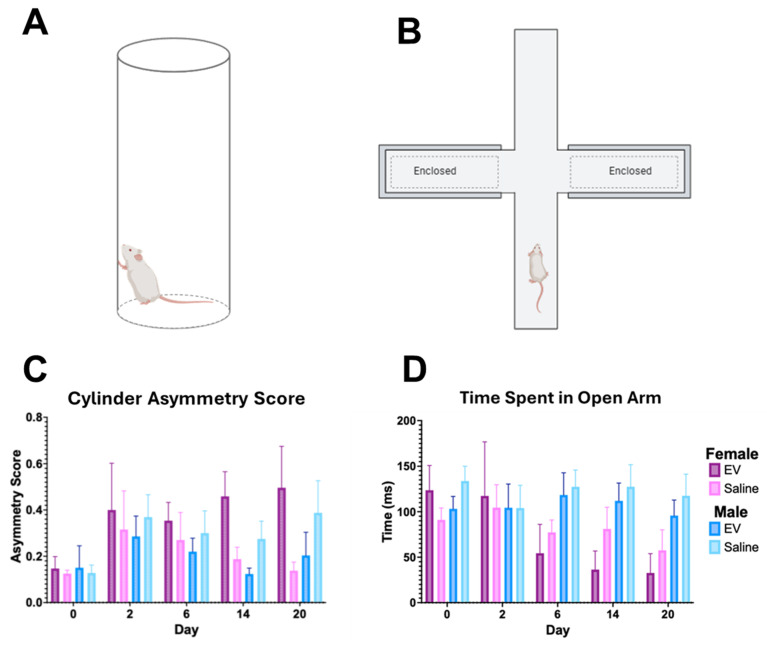
** Forelimb Use Asymmetry and Anxiety-Like Behavior Following EV or Saline Treatment.** (A) Schematics of the cylinder rearing test and (B) elevated plus maze used to assess forelimb use asymmetry and anxiety-like behavior, respectively. (C) Forelimb use asymmetry scores derived from the cylinder rearing test, stratified by sex and treatment group across Days 0, 2, 6, 14, and 20 post-MCAO. (D) Time spent in the open arms of the EPM across the same timepoints, stratified by sex and treatment group. No significant differences between EV and saline treatment groups were detected in either the forelimb asymmetry score or open arm time across the observation period. Statistical significance was assessed using a GLMM with Student's t-test for post hoc pairwise comparisons. No correction for multiple comparisons across outcome measures was applied. *p < 0.05.

The cylinder rearing test was used to assess forelimb use asymmetry on Days 0, 2, 6, 14, and 20 post-MCAO (**Fig. 8A**) as well as the elevated plus maze (**Fig. 8B**). Forelimb use asymmetry scores did not differ significantly between the 3D-EV and saline treatment groups in either sex across the observation period (**Fig. 8C**). These null findings did not produce detectable differences in forelimb use asymmetry or anxiety-like behavior within the timeframe examined, despite the improvements in lesion volume and selected metabolic measures. For the elevated plus maze test, the 3D-EV treated animals initially spent more time in the open arms compared to the control animals. By day 6, however, this behavior declined in the EV group, a change that may reflect either increased anxiety or habituation due to repeated testing. Female saline controls exhibited a similar, albeit slightly lower, reduction. Meanwhile, both male groups do not demonstrate any significant changes in the exploration pattern of the maze over the 21-d period (**Fig. 8D**). The time spent in the open arms of the elevated plus maze did not differ significantly between treatment groups in either sex at any timepoint (**Fig. 8D**).

Body weight was monitored across days 1–10 post-MCAO. In general, male animals demonstrated greater weight loss than female animals. Across both sexes, animal weight gradually recovered over the monitoring period. No significant differences in body weight change were detected between EV and saline treatment groups across the observation period (**Fig. S6**).

## Discussion

This study evaluated the therapeutic efficacy of 3D-EV using ultra-high-field MRI combining T_2_W imaging, DWI, ²³Na MRI, and ¹H-MRS to characterize structural, ionic, and metabolic recovery in male and female rats. Overall, 3D-EV therapy was associated with favorable early trends toward lesion volume reduction, most apparent between Days 1 and 3 post-reperfusion. Female animals generally exhibited more favorable recovery trajectories compared to males regardless of treatment, across imaging techniques, which may suggest that sex-specific biological mechanisms substantially influence post-ischemic repair. These sex-dependent differences were consistent across structural, ionic, and metabolic measures.

Female animals showed trends toward early decreases in ^1^H lesion volume and signal intensity between Days 1 and 3, with the most consistent pattern observed in the 3D-EV-treated cohort. This may suggest attenuation of vasogenic edema within the peri-infarct core 17,46,47. In contrast, male animals exhibited less pronounced reductions. The saline-treated males demonstrated some decreases compared to 3D-EV-treated males at select timepoints. This pattern may reflect greater variability in early injury evolution in this group. These findings suggest that females may exhibit a more favorable early structural trajectory following ischemia, though a larger sample size is needed to make definitive conclusions.

²³Na MRI provided a sensitive metric of ionic dysregulation following ischemia by capturing early changes in sodium accumulation that may reflect impairment of ion transport mechanisms 17,48,49. In both sexes, 3D-EV-treated animals demonstrated trends toward earlier and more sustained reductions in sodium-defined lesion volume beginning at Day 3 out to Day 21. Though these trends were observed in both sexes, they were more consistent and less variable in female animals. This pattern may indicate enhanced recovery of ionic homeostasis following reperfusion, though further investigation is needed 48. Despite the high clearance rate, these trends suggest that EV-mediated effects may be sustained by paracrine modulation of the post-ischemic microenvironment 50-52. That ²³Na MRI was sensitive enough to capture these sex-dependent differences following a single bolus injection, further underscoring its value as a longitudinal biomarker of ionic recovery in preclinical stroke studies 48,53,54.

These findings are consistent with prior work from our group showing that direct hMSC or MSC-EV treatments can attenuate ionic dysregulation during the acute period of ischemia. Direct implantation of hMSC aggregates in male animals found progressive sodium recovery over time 54. Hypoxic preconditioning of conventional 2D MSC-EVs further demonstrated lower lesion variability during the acute phase 29. The present study extends these findings by demonstrating that sodium recovery may differ across sex, with females showing more consistent normalization across both treatment groups 44.

Dysregulation of sodium can lead to rapid cell swelling and cytotoxic edema due to excessive sodium and water influx. This results in the characteristic early reductions in ADC observed following an ischemic insult 53,55. In the present study, ADC values in the ipsilateral hemisphere were lower on Day 1 than in the contralateral hemisphere, consistent with acute cytotoxic injury, and showed progressive recovery across the observation period in both treatment groups. This pattern of early ADC reduction followed by gradual normalization is well established in preclinical stroke models and reflects the evolution from acute ionic dysregulation toward partial stabilization 56.

Sex- and treatment-dependent differences in the recovery were observed over the 21 d period. 3DEV-treated males demonstrated an increase in ADC as early as Day 3. Meanwhile, female animals showed a slower but more sustained normalization that extended through Day 21. These results are consistent with sodium recovery and metabolic patterns and may suggest improvement in ionic, structural, and diffusion metrics. However, these findings did not reach statistical significance and, thus, should be interpreted as trends 57. Additionally, pseudo-normalization, which occurs when ADC values appear to return toward baseline despite ongoing tissue injury, should be taken into consideration when interpreting ADC recovery 58,59. Nonetheless, 3DEV-treated females exhibited trends toward sodium homeostasis and metabolic recovery, further supporting that diffusion recovery may reflect improvement in ADC. However, histological confirmation would be required to fully validate this interpretation.

Previous studies have demonstrated that it is possible to measure ADC under the susceptibility conditions inherent at ultra-high-fields in rodent models 56,60-62. Additionally, early low diffusivity with preserved barrier function has been shown when evaluating diffusion and BBB integrity. Meanwhile, regions with elevated permeability but relatively preserved diffusivity may retain partial viability 56. These technical and mechanistic considerations support the validity of the diffusion findings reported here and provide important context for their biological interpretation. Together, the ADC data complement the structural and ionic findings and reinforce the pattern of sex-dependent differences in post-ischemic tissue recovery.

Single-voxel ¹H-MRS was used to evaluate metabolic alterations related to neuronal integrity, energy metabolism, and membrane dynamics across the 21-day recovery period. Metabolite concentrations were normalized to an external phantom 36, enabling comparison of lactate, NAA, total creatine, and choline.

Lactate remained elevated in male animals across both treatment groups throughout the 21 d period, showing a shift away from oxidative phosphorylation toward anaerobic metabolism. This is consistent with energetic compromise in the post-ischemic period 63,64. In contrast, 3D-EV-treated females showed stable lactate concentrations over the 21 days, potentially reflecting a more effective restoration of oxidative metabolism, although a larger sample size would be needed to make definitive conclusions 64. These findings, along with the sodium recovery and more sustained ADC observed in females, indicate improvement in ionic balance, cytotoxic edema, and mitochondrial function.

NAA serves as a marker of neuronal integrity, and reductions in its concentration reflect loss of viable neurons or impaired neuronal function within the ischemic region 63-67. As expected after ischemia, NAA levels were reduced in the control groups, with males showing the lowest concentrations 29,63. Treated females maintained higher NAA concentrations, a pattern that may be associated with greater neuronal preservation or reduced mitochondrial impairment in this group during early recovery 64. Although these findings point toward early neuronal preservation, NAA measured within a single striatal voxel may not fully capture the broader consequences of stroke. Clinical work has shown that cortical MRS metabolites, including NAA and GLX, do not reliably predict upper extremity motor impairment 68. As a result, cortical NAA alone has limited value as a marker of functional outcome in the later stages of stroke. In contrast, the study by Mazibuko and colleagues showed that NAA measured in the ipsilesional thalamus declined over time and was strongly associated with motor performance at twelve weeks 67. This suggests that metabolic changes in connected regions may provide a clearer picture of network level injury and recovery. NAA measurements in regions, like the thalamus, striatum, and peri-infarct tissue, along with histological confirmation, may offer a more complete assessment of neuronal preservation of treatments.

Total creatine and choline provide additional insight into cellular energy buffering, membrane turnover, and glial activity 69. Both the control and 3DEV-treated male groups showed lower creatine concentrations, consistent with impaired energy buffering after ischemic injury 66. In contrast, 3DEV-treated females maintained their creatine concentrations with a gradual increase over the 21-day period. Meanwhile, female controls, though lower at 24 h post-reperfusion, also showed partial recovery by day 21. Lower choline concentrations were also observed in both male groups. Overall, the females had higher concentrations, with 3DEV-treated females demonstrating the greatest increase. Because choline is sensitive to membrane turnover and inflammation, these elevations may suggest more active glial responses in females 70. These metabolite trajectories parallel findings from a recent clinical diffusion-weighted MRS study, which reported increased diffusivity of choline-containing compounds at 1 month after stroke and increased creatine diffusivity through 3 months, reflecting prolonged microglial activation and later stage astrogliosis 69. Together, these results suggest that choline and creatine shifts reflect glial remodeling and inflammatory activity that extend beyond the acute phase. The more favorable patterns observed in treated females may therefore indicate a more adaptive glial response that complements the neuronal preservation reflected by higher NAA in this group, though direct evidence for this interpretation was not obtained in the present study.

### Impact of Hormonal Milieu on Sex-Specific Recovery and Therapeutic Outcomes

The directional patterns of more favorable recovery observed in female animals across structural, ionic, diffusion, and metabolic measures raise the possibility that circulating ovarian hormones may contribute to these differences, though this mechanism was not directly tested in the present study. Estrogen is known to exert pleiotropic effects on the post-ischemic brain, including modulation of Na⁺/K⁺-ATPase activity, suppression of NF-κB-mediated neuroinflammation, activation of PI3K-AKT pro-survival signaling, and preservation of mitochondrial function 44,71. In the present study, all female animals were young free-cycling adults, and the phase of the estrous cycle at the time of stroke was not controlled. Previous studies have found that infarct size varies across the estrous cycle, with females in metestrus developing larger lesions than those in other phases 72. These observations highlight the importance of including estrous cycle staging as a covariate in future studies.

### Limitations and Future Directions

Several limitations of the present study should be acknowledged. The modest sample sizes limit the statistical power and should be considered when interpreting results that did not reach statistical significance. To further strengthen the interpretation of the MRI and MRS data, histological validation is needed. This would allow for the confirmation of neuronal survival and glial responses. Although no adverse effects were observed following EV administration, this study did not include a comprehensive biosafety assessment, and future studies should include molecular analyses to assess potential toxicity and systemic biodistribution. In regard to sample preparation, the ExtraPEG isolation method has been shown to co-isolate non-vesicular components such as protein aggregates and lipoproteins. While post-processing ultracentrifugation steps reduce this contamination, more stringent approaches such as size exclusion chromatography are being implemented in ongoing work. Relatedly, free USPIO may contribute to the observed GRE hypointensity and cannot be formally excluded as a USPIO-only group was not included. Interpretations of *in vivo* EV localization based on MRI signal should therefore be considered indicative rather than definitive.

## Conclusions

This study demonstrated the utility of combining structural and metabolic imaging modalities including, T_2_-weighted MRI, DWI, and sodium MRI to evaluate treatment efficacy and biological sex differences in recovery following ischemic injury. The results revealed trends towards reduced lesion volumes, progressive ADC normalization, and sodium homeostasis with EV treatment. These findings are further supported by MRS results, which revealed lower lactate concentrations and higher NAA, creatine, and choline concentrations at early time points, especially in females. Behavioral assessments did not reveal significant between-group differences, though open field measures suggested possible locomotor differences at later timepoints. Overall, female animals showed a trend of more favorable recovery than males, though many of these differences did not reach statistical significance. This pattern may reflect the modulatory influence of freely circulating hormones and EV-mediated therapeutic response. Future studies that include the estrous phase as a covariate, repeated dosing strategies and histological validation will be essential to fully characterize the therapeutic potential of EV-based interventions across diverse patient populations.

## Supplementary Material

Supplementary figures.

## Figures and Tables

**Table 1:** Iron Content Measurements for USPIO-Labeled EVs

Cell Group	Concentration	Number of EV in 100 µL	|pg FE|/EV	Average |pg FE/EV|
Unlabeled 1	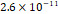	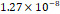	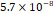	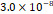
Unlabeled 2	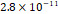	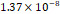	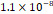	
Unlabeled 3	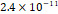	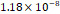	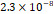	
Labeled 1	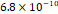	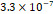	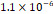	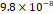
Labeled 2	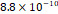	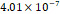	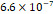	
Labeled 3	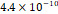	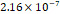	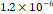	

**Table 2:** Average ipsilateral and contralateral ADC values (×10⁻³ mm²/s) for male and female rats following EV or saline treatment.

		Apparent Diffusion Coefficient ( 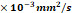 )							
		Contralateral				Ipsilateral			
		EV		Saline		EV		Saline	
Sex	Day	Mean	STD	Mean	STD	Mean	STD	Mean	STD
**Female**	**1**	0.98	0.32	0.92	0.20	0.76	0.26	0.63	0.12
	**3**	0.91	0.21	0.82	0.13	0.82	0.20	0.79	0.19
	**7**	0.86	0.06	0.83	0.15	0.86	0.11	0.82	0.15
	**21**	0.85	0.24	0.83	0.15	0.88	0.23	0.85	0.17
**Male**	**1**	0.78	0.32	1.05	0.22	0.64	0.21	0.82	0.25
	**3**	0.94	0.30	1.14	0.10	0.93	0.39	1.03	0.14
	**7**	0.91	0.12	1.10	0.23	0.85	0.16	1.05	0.26
	**21**	0.98	0.32	1.06	0.10	1.04	0.23	1.00	0.19

## Data Availability

The data supporting the findings of this study are available from the corresponding author upon reasonable request.

## Abbreviations

MRI: Magnetic Resonance Imaging
T_2_W: T_2_-Weighted
DWI: Diffusion-Weighted Imaging
DWI-EPI: Diffusion-Weighted Imaging Echo-Planar Imaging
ADC: Apparent Diffusion Coefficient
¹H-MRS: Proton Magnetic Resonance Spectroscopy
²³Na MRI: Sodium-23 Magnetic Resonance Imaging
CSI: Chemical Shift Imaging
GRE: Gradient-Recalled Echo
RARE: Rapid Acquisition with Relaxation Enhancement
sLASER: Semi-Adiabatic Localization by Adiabatic Refocusing
VAPOR: Variable Power Radiofrequency Pulses with Optimized Relaxation Delays
SNR: Signal-to-Noise Ratio
ROI: Region of Interest
FID: Free Induction Decay
RF: Radiofrequency
TE: Echo Time
TR: Repetition Time
NAA: N-Acetylaspartate
Lac: Lactate
Cre: Creatine
Cho: Choline
TSP: Trimethylsilylpropanoic Acid
GABA: γ-Aminobutyric Acid
ATP: Adenosine Triphosphate
MSC: Mesenchymal Stem Cell
hMSC: Human Mesenchymal Stem Cell
EV: Extracellular Vesicle
3D-EV: Three-Dimensional hMSC Aggregate-Derived Extracellular Vesicles
MSC-EV: Mesenchymal Stem Cell-Derived Extracellular Vesicles
USPIO: Ultrasmall Superparamagnetic Iron Oxide
NTA: Nanoparticle Tracking Analysis
TEM: Transmission Electron Microscopy
CCM: Complete Culture Medium
ULA: Ultra-Low Attachment
PEG: Polyethylene Glycol
PBS: Phosphate-Buffered Saline
PFA: Paraformaldehyde
FBS: Fetal Bovine Serum
miRNA: MicroRNA
PI3K: Phosphoinositide 3-Kinase
AKT: Protein Kinase B
NF-κB: Nuclear Factor Kappa B
VEGF: Vascular Endothelial Growth Factor
GPER1: G-Protein Coupled Estrogen Receptor 1
MCAO: Middle Cerebral Artery Occlusion
MCA: Middle Cerebral Artery
tMCAO: Transient Middle Cerebral Artery Occlusion
ICA: Internal Carotid Artery
ECA: External Carotid Artery
CBF: Cerebral Blood Flow
GLMM: Generalized Linear Mixed-Effects Model
LS Means: Least Squares Means
SD: Standard Deviation
SEM: Standard Error of the Mean
EPM: Elevated Plus Maze
OFT: Open Field Test
FDA: Food and Drug Administration
tPA: Tissue Plasminogen Activator
ACUC: Animal Care and Use Committee
NIH: National Institutes of Health
NHMFL: National High Magnetic Field Laboratory
α-MEM: Alpha-Minimum Essential Medium
