# Supplementary material for: Ultra-high-field magnetic resonance imaging reveals sex-specific recovery after ischemic stroke following treatment with three-dimensional human mesenchymal stem cell–derived extracellular vesicles
Source: Theranostics. 2026 Jun 17;16(13):7698–714. doi: 10.7150/thno.129188 (PMC13295759; doi:10.7150/thno.129188)
Supplement: Supplementary file 1 — Supplementary figures. [file thnov16p7698s1.pdf]

Supplementary Materials

Ultra-high-field magnetic resonance imaging reveals sex-specific recovery after ischemic stroke following treatment with three-dimensional human mesenchymal stem cell–derived extracellular vesicles

Jamini Bhagu [a,b], Arshia Arbabian [a,b], Thurston Da Vitoria Lobo [a,b], Katherine Martinez [a,b], Dayna L. Richter [a,b], Hannah Bryant [a,b], Malathy Elumalai [a], Yan Li [a,b]*, Samuel C. Grant[a,b]*

[a] *National High Magnetic Field Laboratory, Florida State University, Tallahassee, Florida, USA*

[b] *Department of Chemical & Biomedical Engineering, FAMU-FSU College of Engineering, Florida State University, Tallahassee, Florida, USA*

***Corresponding authors:**

Dr. Yan Li: 2525 Pottsdamer St., Tallahassee, FL 32310, USA, Tel: 850-410-6320; Fax: 850-410-6150; email: yli4@fsu.edu.

ORCID: Yan Li: 0000-0002-5938-8519

Dr. Samuel C. Grant, email: grant@magnet.fsu.edu. ORCID is: 0000-0001-7738-168X

Supplementary Figures: 6

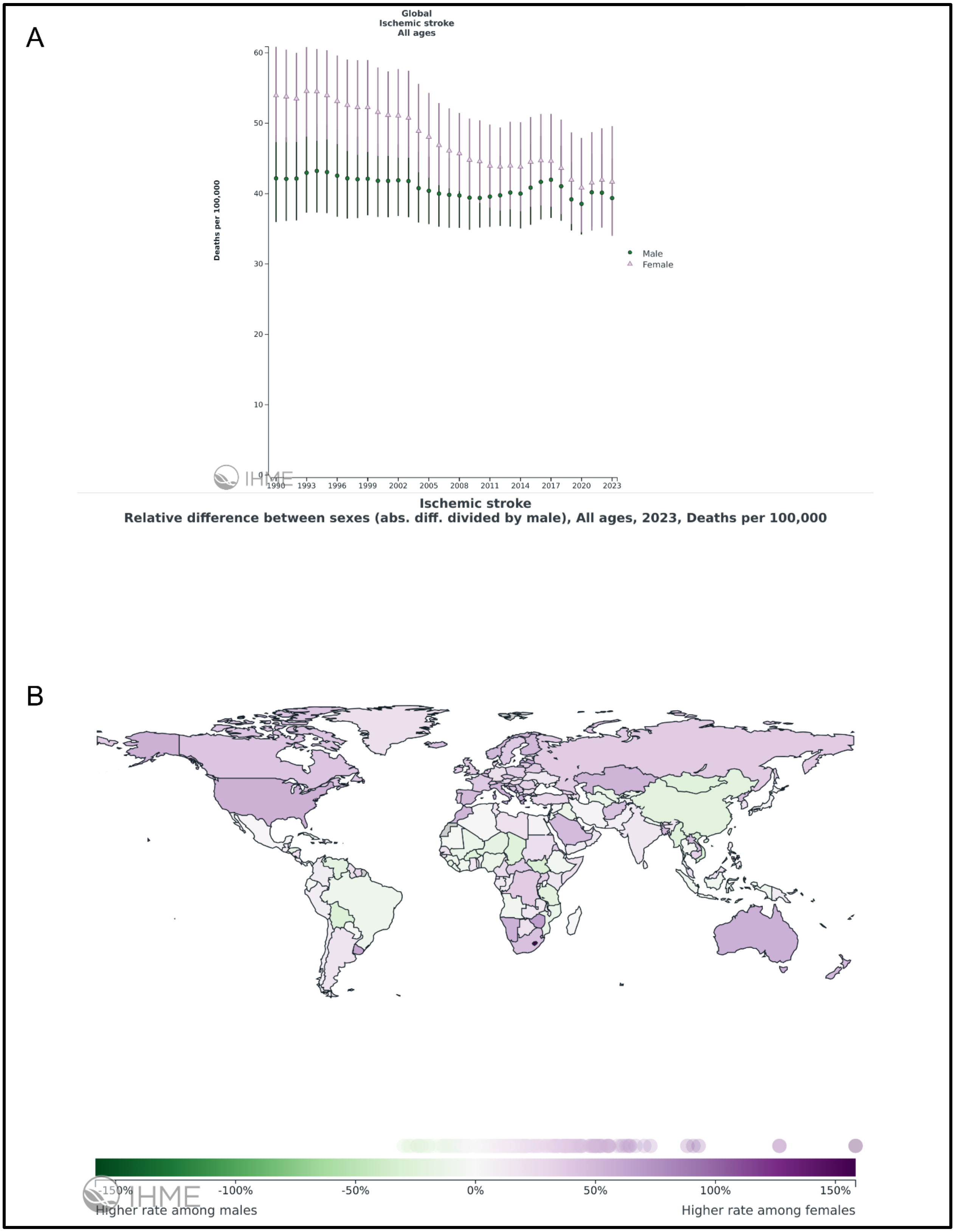
A
Global
Ischemic stroke
All ages
Deaths per 100,000
0
10
20
30
40
50
60
1990
1993
1996
1999
2002
2005
2008
2011
2014
2017
2020
2023
Male
Female
IHME
Ischemic stroke
Relative difference between sexes (abs. diff. divided by male), All ages, 2023, Deaths per 100,000
B
-150%
-100%
-50%
0%
50%
100%
150%
Higher rate among males
Higher rate among females
IHME

Supplementary Figure S1. Global ischemic stroke mortality and sex differences. (A) Globally standardized deaths per 100,000 for ischemic stroke from 1990 to 2023. The points represent annual estimates and vertical lines indicate uncertainty intervals. (B) Relative difference in ischemic stroke mortality between females and males. Positive values indicate higher mortality among females. Negative values indicate higher mortality among males. Data source: Global Burden of Disease (GBD) Study, Institute for Health Metrics and Evaluation (IHME), University of Washington. Generated using the IHME GBD Compare and GBD Sex Differences visualization tools. Available from: http://vizhub.healthdata.org/gbd-compare/sex-differences. Accessed March 2, 2026. Screenshot reproduced for non-commercial academic use with attribution under the Creative Commons Attribution-NonCommercial-NoDerivatives 4.0 International License (CC BY-NC-ND 4.0): https://creativecommons.org/licenses/by-nc-nd/4.0/. No changes were made to the original visualization except resizing/formatting for manuscript presentation.

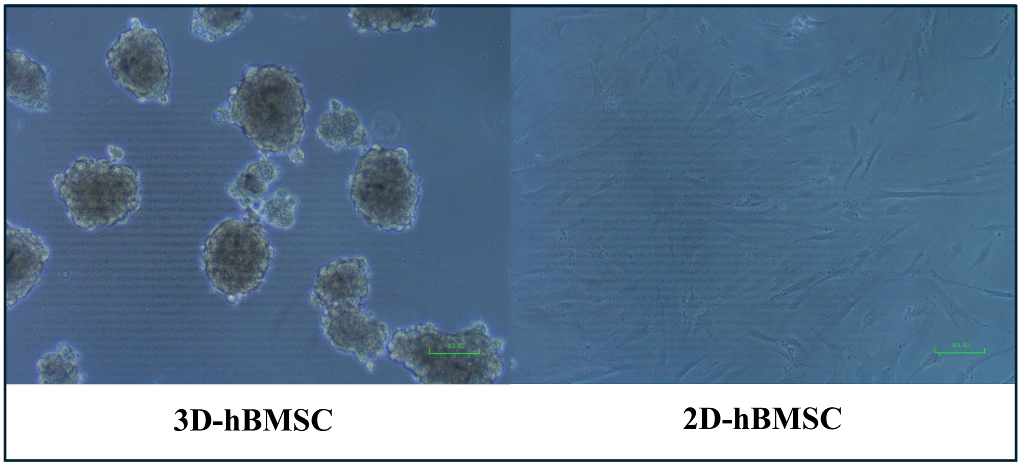

Supplementary Figure S2. **Morphology of human bone marrow MSC (hBMSC) cultured under 2D and 3D conditions.** Left**:** The 3D-hBMSC spheroids are characteristic of 3D dynamic growth. Right**:** 2D-hBMSCs showing the characteristic elongated, spindle-like morphology. The images were collected with bright-field microscopy. Scale bar = 100 µm.

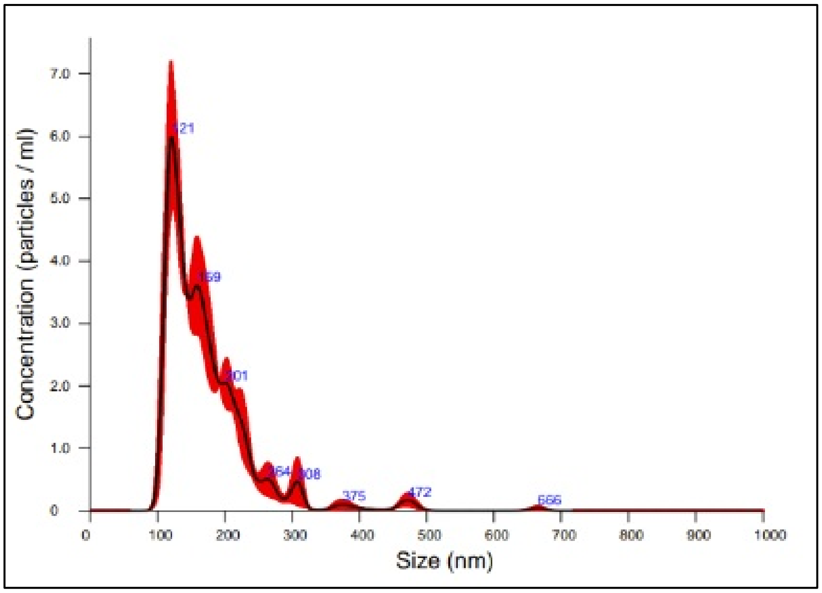

Supplementary Figure S3. Nanoparticle Tracking Analysis (NTA) of EVs. Representative EV sample with a peak near 120 nm. The red curve represents the mean distribution.

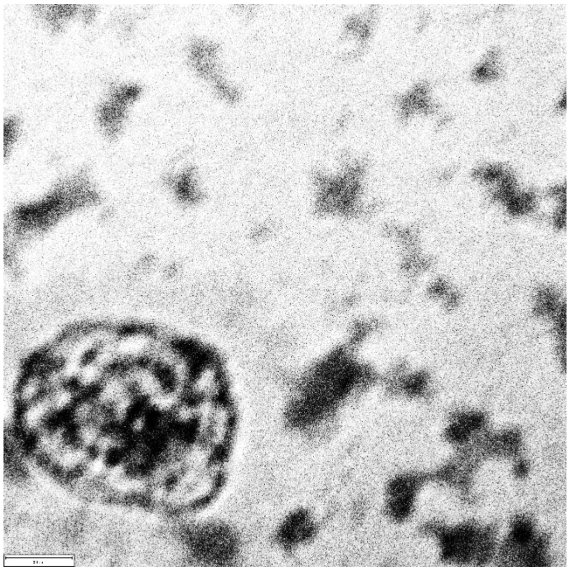

Supplementary Figure S4. Transmission electron microscopy image of USPIO labeled EVs.
Scale bar: 60 nm.

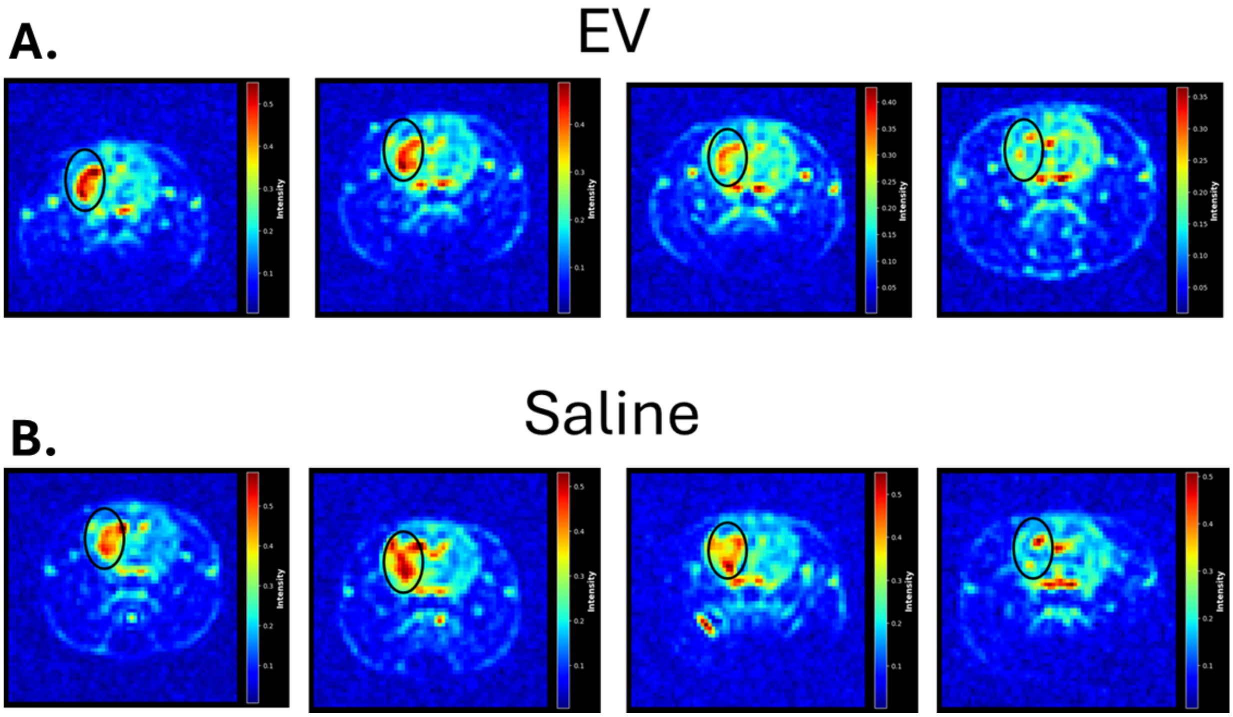

Supplementary Figure S5: Longitudinal Changes in ^{23}Na MRI Signal (Male). 3D EV-treated (A) and control (B) male animals, after days 1, 3, 7, and 21 post-MCAO. The black ellipse delineates the ischemic lesion.

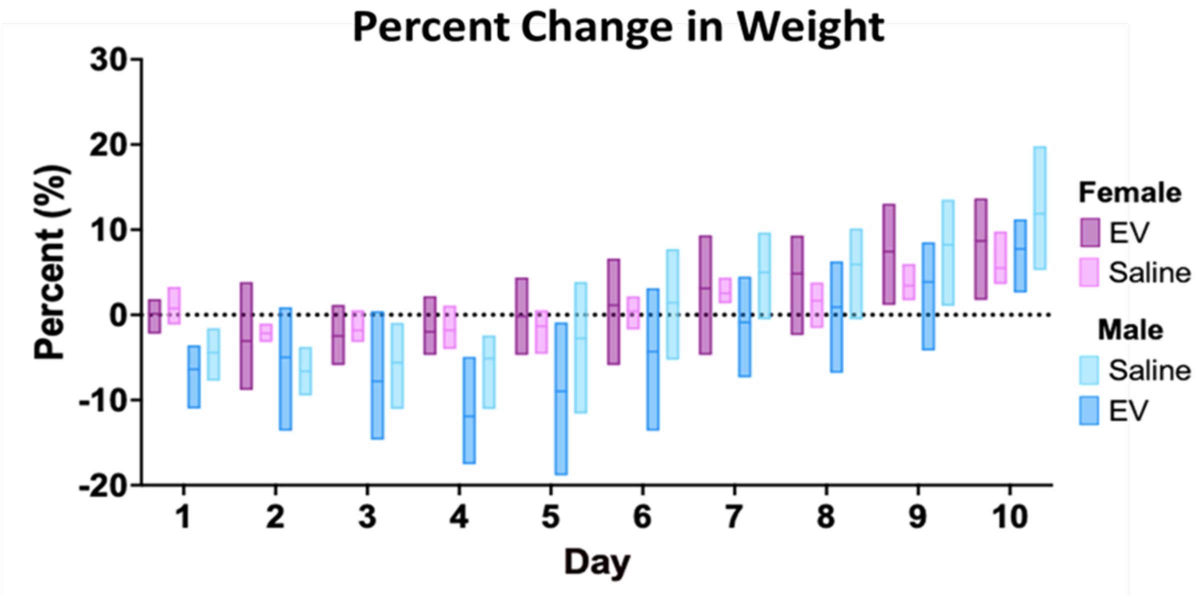

Supplementary Figure S6: Percent Change in Body Weight Following MCAO. Percent change in body weight post-MCAO. Data are presented as mean ± SEM. Statistical analysis was performed using a generalized linear mixed-effects model. The fixed effects include Day, Treatment, and the Day × Treatment interaction. Student's t-test was performed for pairwise comparisons.
